# Computational Logistics for Container Terminal Handling Systems with Deep Learning

**DOI:** 10.1155/2021/5529914

**Published:** 2021-04-26

**Authors:** Bin Li, Yuqing He

**Affiliations:** ^1^School of Mechanical & Automotive Engineering, Fujian University of Technology, Fuzhou 350118, China; ^2^School of Transportation, Fujian University of Technology, Fuzhou 350118, China

## Abstract

Container terminals are playing an increasingly important role in the global logistics network; however, the programming, planning, scheduling, and decision of the container terminal handling system (CTHS) all are provided with a high degree of nonlinearity, coupling, and complexity. Given that, a combination of computational logistics and deep learning, which is just about container terminal-oriented neural-physical fusion computation (CTO-NPFC), is proposed to discuss and explore the pattern recognition and regression analysis of CTHS. Because the liner berthing time (LBT) is the central index of terminal logistics service and carbon efficiency conditions and it is also the important foundation and guidance to task scheduling and resource allocation in CTHS, a deep learning model core computing architecture (DLM-CCA) for LBT prediction is presented to practice CTO-NPFC. Based on the quayside running data for the past five years at a typical container terminal in China, the deep neural networks model of the DLM-CCA is designed, implemented, executed, and evaluated with TensorFlow 2.3 and the specific feature extraction package of tsfresh. The DLM-CCA shows agile, efficient, flexible, and excellent forecasting performances for LBT with the low consuming costs on a common hardware platform. It interprets and demonstrates the feasibility and credibility of the philosophy, paradigm, architecture, and algorithm of CTO-NPFC preliminarily.

## 1. Introduction

Container terminals are the important hub nodes of the global logistics network, and the container terminal handling system (CTHS) has typical characteristics of nonlinearity, hierarchy, dynamic, timeliness, randomness, context-sensitivity, coupling, and complexity (NHDT-RCCC) [1, 2]. As a result, the layout programming, process design, job planning, task scheduling, resource allocation, and collaborative decision of CTHS, which are abbreviated as PDP-SAD, all are of nondeterministic polynomial completeness (NPC), and these have been the focus and difficulty of the theoretical research and engineering practice for operations research and logistics industry.

A lot of scholars have a number of discussions on the PDP-SAD in CTHS with various methods, such as layout design [3], berth allocation [4], quay crane planning [5], yard crane scheduling [6], and integrated scheduling [7]. Nevertheless, a large number of difficult problems in CTHS remain to be solved and new ones emerge one after another with the rapid development of the port and waterway industry. Consequently, a very obvious trend is that more and more automated and intelligent methods and means are applied to CTHS gradually. Kavoosi et al. proposed the augmented self-adaptive parameter control strategy for evolutionary algorithm to solve berth scheduling problem [8]. Rekik and Elkosantini suggested a multiagent approach for the reactive and decentralized control of container stacking in an uncertain and disturbed environment [9]. More importantly, the machine learning and deep learning both have been applied to the PDP-SAD at container terminals slowly, such as container relocation problem [10] and container premarshalling problem [11].

For smoothing and overcoming the NHDT-RCCC of CTHS, this paper tries to integrate computational logistics and deep learning to discuss and explore the running of CTHS by the refinement, generalization, transferring, unification, integration, and fusion of problem-oriented computation (RGT-UIF-PoC). The combination is clearly different from the classical research methodology and existing engineering solution to CTHS, and it conforms to the current research and development trend of intelligent logistics systems as well. More critically, the combination of computational logistics and deep learning is supposed to construct container terminal-oriented neural-physical fusion computation (CTO-NPFC) based on the abstraction and automation of computation, which is intended to provide intelligent decision support compound engine for the PDP-SAD of CTHS with soft real-time constraint conditions both at the tactical level and on the strategic level.

The reminder of this paper is organized as follows: Section 2 provides the overview of computation logistics and deep learning and clarifies the reasons for combining the two.  Section 3 presents CTO-NPFC to design and implement the container terminal-oriented logistics generalized computing automation and intelligence and proposes the deep learning model core computing architecture (DLM-CCA) for liner berthing time (LBT) prediction. A real case study of CTHS, which covers LBT forecasting experiments and performance evaluation by CTO-NPFC, is discussed at length in Section 4.  Section 5 concludes the paper with some discussions and extensions.

## 2. Computational Logistics and Deep Learning

### 2.1. Computational Logistics and Practical Philosophy

Over the past 15 years, we have been focusing on the discussion and exploitation of computational logistics. The initial definition of computation logistics was proposed by Bin Li on the 54th IEEE Conference on Decision and Control (CDC 2015) in 2015 based on a decade of exploration since 2006 [12], which was briefly stated as follows.

Computational logistics involves the programming, designing, implementation, testing, and evaluation of complex logistics systems (CLS) and then includes the planning, controlling, scheduling, and decision-making for the related logistics service procedure in the various administrate levels, which forms a unified and quantitative universal approach for design, construction, execution, management, and improvement of CLS by the extraction, transformation, and application of basic concepts, fundamental principles, decision framework, control mechanism, and scheduling algorithms in computer science and automatic control theory in accordance with theory of computation and similarity theory.

From the above definition, it is concluded that the computational logistics is significantly different from the traditional methods for CLS that cover mathematic programming, system simulation, intelligent optimization, and simulation-based optimization [13–15]. The theoretical origins of computational logistics mainly include computational thinking, computational lens, theory of computation, and great principles of computing [16], which are collectively referred to as 4CTTLP.

It is critical that the computational logistics derives from practice and in turn serves practice. The former practice means the exploration and application of 4CTTLP in the field of computer and control science and engineering, and the latter practice signifies the exploitation and development of 4CTTLP in the domain of CLS. The former focuses on the computation in the cyberspace, and the latter discusses the issues of the computation in the physical world emphatically. The nature of computation builds the bridges between the computation in the cyberspace and the counterparts in the physical world and then gives a unified abstract and automated perspective of computation for both. To some extent, the study of computational logistics aims at providing an insight into the intensions and characteristics of the computations occurring in the physical world. Then, the essence, connotation, and extension of computation can be further clarified in the context of CLS. As a result, the RGT-UIF-PoC is the core principles and practical philosophy of computational logistics.

Actually, in the absence of automated container terminals, some scholars have applied computational methods and control theory to the PDP-SAD of the traditional CTHS over the past decade or so, which exert the philosophy of computational logistics unconsciously. The works utilize feedback control [17], queue-based local scheduling [18], predictive allocation [19], parameter tuning and control [20], and multistage global coordination [21] into the scheduling and decision at container terminals. The above discussions lay a good foundation for the establishment, development, and demonstration of computational logistics.

### 2.2. Deep Learning for Scheduling and Decision

Deep learning originates from the discussion and exploration of artificial neural network (ANN) and deep neural networks (DNN), and it is a deep machine learning model [22]. The deep learning executes a series of nonlinear transformation to study how to automatically extract the multilayer characteristics from the original data, and it has been widely applied in the field of image recognition, speech recognition, natural language processing, drug discovery, and so on [23].

However, the deep learning is rarely applied to the programming, planning, scheduling, control, and decision-making of complex operating systems for the moment. At the same time, some preliminary discussions are beginning to be done, especially based on ANN and DNN. The deep learning is tentatively applied for shipping container code localization and recognition [24], machine conditions prediction [25], logistics delivery demand [26, 27], and railway transportation fault diagnosis [28]. The above works are a preliminary attempt to apply deep learning to complex operating systems, especially for logistics service hubs. Nevertheless, there are still lots of works to be done on scheduling and decision making for CLS at the strategy and tactical level.

### 2.3. Tentative Combination for Container Terminal Handling Systems

Within the conceptual framework of computational logistics, the information computing in the traditional sense does not meet the requirements of the PDP-SAD at container terminals, and it is only the state, activity, and transaction mapping between the information space and the physical world. The pure information management and process mirroring is not the wing plane of the computational logistics because it does not provide intelligence decision support for the running of CTHS.

However, the abstract models and automation machines of CTHS by computational logistics really need the problem-oriented, process-oriented, and scenario-oriented machine intelligence. The deep learning can take this responsibility for CTHS, and it is based on the ANN or DNN that has the excellent ability of self-learning, self-organization, self-adaptation, and strong nonlinear function approximation [23]. So the deep learning is just about the following aircraft of computational logistics.

For the PDP-SAD at container terminals, the deep learning is expected to conduct the pattern recognition, clustering analysis, regression forecast, and performance evaluation to drive the planning, scheduling, deployment, execution, and commissioning of CTHS. At present, the deep learning is seldom applied in the control and decision making of CLS. The computational logistics establishes a sound and solid abstraction and automation foundation of CTHS for the application of deep learning.

In reality, we try to combine computational logistics and deep learning tentatively to figure out the PDP-SAD in CTHS from the perspective of computation. The scenario-oriented learning is a prominent characteristic of the combination. The computational logistics learns from the computer science and control engineering by 4CTTLP, and the deep learning can execute self-learning and self-evolution from the operational log robustly. The two are supposed to complement each other to overcome NHDT-RCCC together.

## 3. Logistics Generalized Computing Automation and Intelligence for Container Terminals

### 3.1. Logistics Generalized Computation for Container Terminal Hubs

The discussion and concerning focus of computational logistics is the generalized computation in the physical world primarily, especially for the CTHS. The essence of computation is every process. More specifically, the substance of computation is a mechanical motion that is driven by energy and controlled by instructions in a narrow sense.

It is obvious that the internal behaviors of CTHS achieve a higher level of compliance with the substance of computation even in a narrow sense. The gaps between the computation in computer systems and the corresponding parts in CTHS are mainly due to the huge differences in processing time scales and spatial dimensions. Those directly lead to the differences in the aspects of computational constraints and optimization objectives. Nevertheless, those have no impact on the comparability, similarity, intercommunity, and uniformity in terms of abstraction and automation between computer systems and CTHS.

According to the essence and connotation of computation, the internal behaviors of CTHS can be abstracted as a kind of generalized computation that is the container terminal-oriented logistics generalized computation (CTO-LGC). On the condition that the various container units are abstracted as the logistics generalized computation alphabet that have been defined in our previous studies [29], the CTO-LGC covers the positioning, relocation, mapping, accessing, shifting, transferring, handling, switching, and routing of container logistics units (PRM-AST-HSR), which construct the instruction set architecture for CTO-LGC in effect. The CTO-LGC is the critical application of computational logistics for CTHS. On the one hand, it is the synthesis and abstraction of container terminal handling process and physical behaviors. On the other hand, the CTO-LGC is the most direct reflection of the computability of CTHS [16], and it establishes the foundation of evaluating logistics generalized computational complexity at container terminals [29].

### 3.2. Logistics Generalized Computation Automation and Intelligence

**Figure 1:**
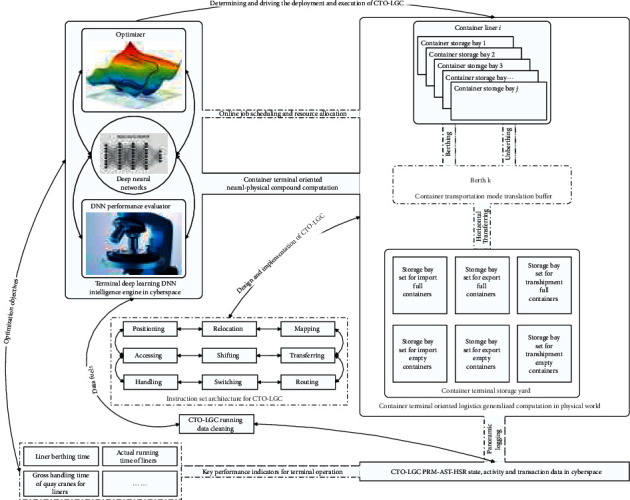
Container terminal-oriented neural-physical fusion computation preliminary sketch.

The above CTO-LGC provides a solid foundation for further discussion by abstraction, and the CTO-LGC governs the center position of the computational logistics for CTHS. The automation and intelligence of CTO-LGC are the next focus of computational logistics. By the combination and integration of computational logistics and deep learning, we make a definition of CTO-NPFC based on the nature of computation and algorithms to discuss the issue whose conceptual framework is illustrated in Figure 1.

The CTO-NPFC integrates and unifies the DNN computing process and CTO-LGC physical process to design, implement, and execute the automation and intelligence of CTO-LGC that is intended to achieve the agile, efficient, and robust operation of CTHS by transferring and learning. The CTO-NPFC is the extension and expansion of cyber-physical systems in the context of intelligent logistics systems for CTHS. The CTO-NPFC is intended to provide an insight, perspective, framework, paradigms, patterns, and algorithms for the PDP-SAD of CTHS.

The solution and practice of CTO-NPFC are challenging and crucial for the automation and intelligence of CTO-LGC. For the automation of CTO-LGC, the instruction set architecture for CTO-LGC establishes an abstract perspective and automation mechanism for the programming tactics and algorithm design of PDP-SAD at container terminals. For the intelligence of CTO-LGC, the deep learning must provide the powerful and flexible machine intelligence to improve the running level of CTHS. Now we focus on the deep learning engine of CTO-NPFC to explore its computing architecture, core principles, key components, design paradigms, and adaptive training, which establishes the underlying operational architecture and core algorithms of CTO-NPFC.

### 3.3. Deep Learning Model Core Computing Architecture for Liner Berthing Time Prediction

According to the above CTO-NPFC preliminary sketch, it is concluded that the berth has essential function and influence in the operation of CTHS because it is the container transportation mode translation buffer that is also the most valuable service resource in CTHS. As the berth serves for the calling container ships that are the service center objects at container terminals, the liner berthing time (LBT) is crucial to both of terminals and carriers, especially for berth allocation and quay crane scheduling. In reality, the prediction and evaluation of LBT determine the operation schedule and resource allocation of CTHS in significant measure. To a large extent, the LBT can determine and forecast carbon emission efficiency so as to facilitate the sustainable development of container terminals.

**Figure 2:**
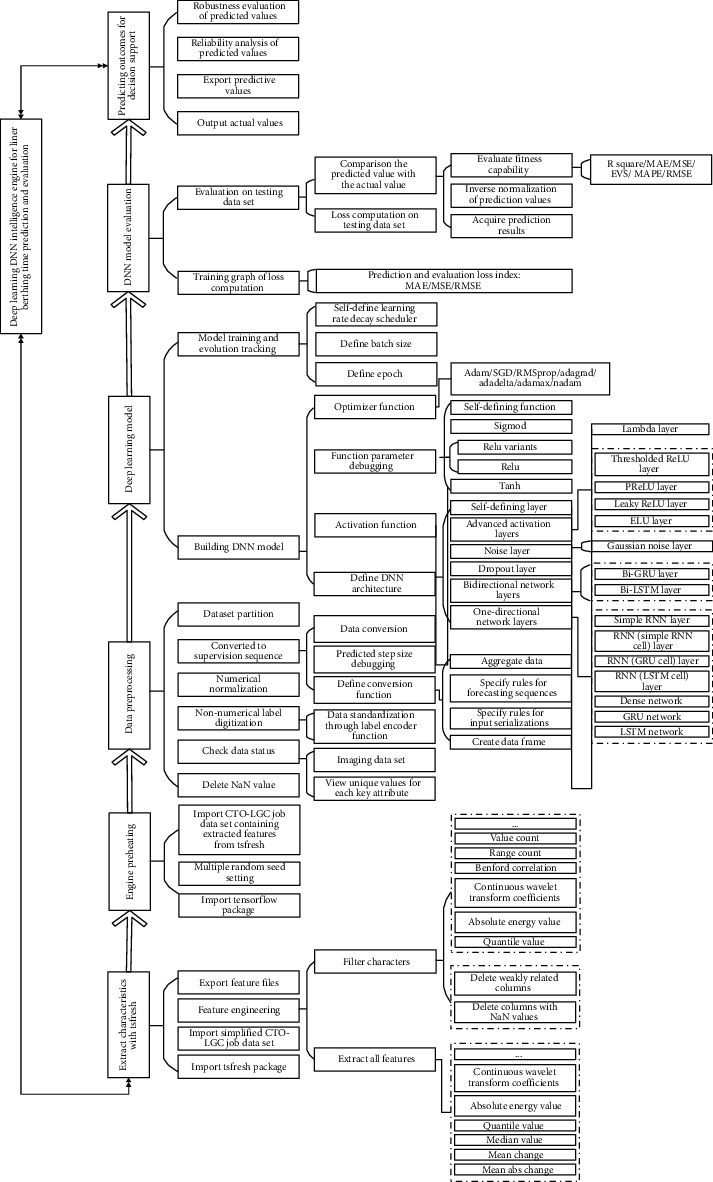
Deep learning model core computing architecture for liner berthing time prediction.

Hence, we define the deep learning model core computing architecture (DLM-CCA) for LBT forecasting, which is demonstrated by Figure 2. As a matter of fact, the DLM-CCA not only is for the prediction of LBT, but also is appropriate for the forecasting of the other key performance indicators of CTHS. Through a lot of experiments, the DLM-CCA in Figure 2 has been proven to be an agile, efficient, robust, tailorable, and portability computing architecture and design paradigm for the various CTHS.

The DLM-CCA is a lightweight deep learning intelligent engine with the perfect property of configurability, flexibility, agility, robustness, and high efficiency, which is good for applying to the intelligent decision support for CTHS not just the prediction of LBT. The DLM-CCA actually consists of six components that are time series feature extraction module, deep learning engine preheating module, liner service data preprocessing, deep learning DNN architecture, DNN model evaluation, and predicting outcome for decision support. The six modules construct a six-phase deep learning pipeline, and they can be further refined into 21-stage sequential process, and those can be defined, debugged, configured, and tailorable, respectively. Now we elaborate on the points of the DLM-CCA.

### 3.4. Deep Learning Kernel of Liner Berthing Time Prediction

The above DLM-CCA is an important component of CTO-NPFC and is the machine intelligent engine of CTHS for pattern recognition, clustering analysis, regression prediction, and so on. The following key points make up the deep learning kernel of LBT prediction.

For one thing, the time series feature extraction of the LBT is a crucial step for the CTO-NPFC, which is based on a Python package that is time series feature extraction on basis of scalable hypothesis tests named as tsfresh. The tsfresh has a low computational complexity and allows starting on a problem with only limited domain knowledge available [30]. Moreover, the tsfresh implements standard application programming interfaces of time series and machine learning libraries and is designed for both exploratory analyses as well as straightforward integration into operational data science applications [31]. In recent years, the tsfresh has been widely applied in many fields, such as predicting the smart grid stability [32], artificial intelligence for cloud storage array operations [33], and the pressure transients of pipe networks in industries and water distribution systems [34]. However, the tsfresh has not been applied to the PDP-SAD of CLS. We integrate the tsfresh into the prediction of LBT to support the scheduling and decision of CTHS. This is a significant, meaningful, and workable attempt to the complex operating systems.

Furthermore, the definition of deep learning model that is no other than the DNN architecture is the deep learning kernel of LBT forecasting. As a principal foundation, the liner berthing time series and relevant liner handling data are integrated and converted into the specific supervised training data frame on the basis of data numeralization, standardization, and normalization in the first place. The given terminal quayside running data frame set is going to be divided into three parts: training set, validation set, and test set, and the ratio of the three can be adjusted and modified according to the actual situations and decision requirements. The DNN architecture is established with the diverse nervous layers flexibly. Those mainly cover unidirectional neural network layer and bidirectional neural network layer. The former includes long short-term memory (LSTM) network layer, gated recurrent unit (GRU) network layer, dense network layer, simple recurrent neural network (RNN) network layer, and RNN cell network layer. The latter contains principally the bidirectional-LSTM and bidirectional-GRU. In addition, the dropout layer, noise layer, advanced activation layer, and self-defined Lambda network layer are all the important components of DLM-CCA. According to the different terminal layout, handling technology, equipment configuration, and task load, the DLM-CCA can be customized and clipped efficiently and expeditiously.

Besides, the components composition, parameter setting, and running configuration are of vital importance for the performance of DLM-CCA. For the prediction of LBT, the Tanh is propitious to be as activation function, and the Adam is suitable for holding the post of optimizer through a lot of experimentations. Meanwhile, the training frequency of DNN model, batch size of single model training, and model learning rate all have important influences on the performance of the DLM-CCA. All of these require a mass of debugging when the DLM-CCA comes to specific objects and applications. According to the practical application, the DLM-CCA shows stable and efficient forecasting performance once the DNN model has been trained and debugged successfully for the given CTHS.

## 4. Computational Experiments

### 4.1. Case Scenario and Experimental Platform

A regional and traditional container terminal hub in China is the target object for the discussion of the CTO-NPFC. There are five deep water berths along terminal quayside, and 12 quay cranes with the four different activation parameters and handling specifications are deployed along quayside. The annual container throughput of terminal is about two million twenty-foot equivalent units (TEUs). About 75 to 85 percent of the visiting container ships are attached to the domestic trade routes in China as appropriate, and the other liners serve for international trade routes. It is a very typical large-scale container terminal by the east coast of China.

The above DLM-CCA is a lightweight single target computing architecture that has high self-learning computation efficiency. Moreover, it requires less hardware computing platform. The experimental platform can be briefly stated as follows. The hardware platform is mainly based on the central processing unit (CPU) of the Intel Core Intel i7-9750H and the graphics processing unit (GPU) of the NVIDIA GeForce GTX 1660 Ti whose computing capability is 7.5 for compute unified device architecture (CUDA). Besides, the main memory is 24 GB, and the video memory is 6 GB. The software platform is primarily based on the TensorFlow 2.3 for GPU and the tsfresh package whose version is 0.170. The whole DLM-CCA is designed, implemented, and executed by Python 3.7.3.

### 4.2. Loading Task Set Evaluation of Container Terminal-Oriented Logistics Generalized Computation

Because the domestic routes container liner occupies the vast majority of calling container ships that more than three quarters liners are used for internal trading container transportation for any given year, we select the corresponding liner calling, berthing, and handling log as the loading task set of the DLM-CCA to explore the prediction of LBT. In reality, as the LBT is also the most important indicator to measure CTO-LGC, the LBT of domestic liner services really have a more important position and function than the ones in international service for the running of CTHS in China both in the short term and in the long term. It can be seen from the growth trend of container throughput of Chinese ports recently, especially since the COVID-19 outbreak.

**Figure 3:**
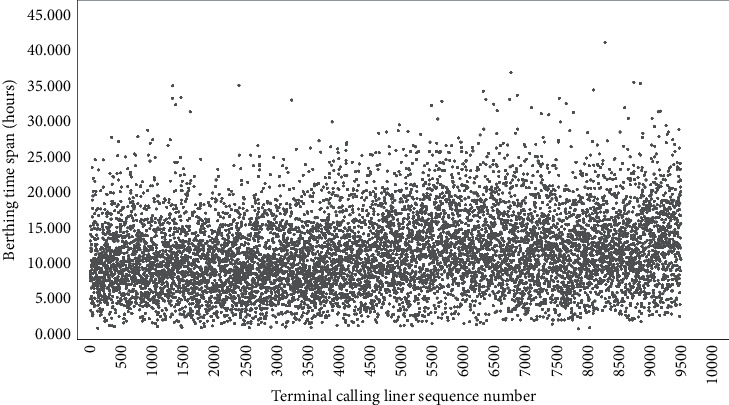
Distribution preliminary sketch of visiting liners berthing time.

According to the field research and data collection at the container terminal, it is almost impossible to be more than 36 hours for the LBT of domestic trade routes in practice. In a recent five-year span, there are 9433 effective job records of calling liners after data cleaning, which are identified as the absolute foundation for the DLM-CCA and called after quayside running mirror for liner berthing time (QRM-LBT). The QRM-LBT is a quantitative random job testing for CTO-LGC, which has been elaborated in our previous work [15]. The general conditions of LBT may be demonstrated by Figure 3 and Table 1 generally. In Table 1, the SD means standard deviation, and the same is true in subsequent statistical tables. It is easy to infer that the practical LBT is highly volatile and stochastic, which makes the prediction of LBT very intractable.

Furthermore, there is a distinguishing feature in the QRM-LBT. It is that the overwhelming majority of LBT within 24 hours goes as high as 9243 items, and the proportion in totality reaches up to 97.986 percent. Besides, there are 158 running records of LBT that is between 24 hours and 30 ones, which accounts for 1.675 percent of all records. There are 30 running records of LBT that is between 30 hours and 36 ones, which accounts for 0.318 percent of QRM-LBT. There are only two records of LBT between 36 hours and 42 ones whose proportion is almost negligible. In other words, only 190 items of LBT are above 24 hours, but those exert a random perturbation to the prediction of LBT. Both make the LBT forecasting a huge challenge. Nevertheless, it possesses the important theoretical significances and precious practical values.

### 4.3. Critical Feature Extraction of Liner Berthing and Handling Operational Log

The Python package of tsfresh is designed to extract characteristics from time series [29] and avoids the time-consuming problem caused by manual statistical calculation and greatly speeds up the progress of data set feature processing. The core application of the tsfresh is divided into two steps. Now we discuss these two steps in detail.

For one thing, the tsfresh can acquire a comprehensive number of features by doing statistical analysis. All feature calculators are contained in the model of feature extraction. By inputting time series data to calculator, we can get the values of a series of characteristics, which are used in subsequent programs. In the applied tsfresh of 0.17.0 version, 72 features computational methods including absolute energy value are provided. In the setting phase of feature extraction, we try to reserve the default options because the tsfresh has provided a set of reasonable parameters for all feature calculators in most instances. We can also make additional special settings on a few feature extractors to meet the computing needs of the given types of features. When applying the feature extraction module, only a small minority of features are found to be relevant in the practice, which usually revolve around a core set of indicators for the target of prediction as a rule of thumb. Consequently, we can extract key features to avoid calculating unnecessary function values to save time. Through the implementation of feature extraction, multiple feature indexes including mean absolute change, mean change, and quantity value can be obtained precisely.

For another, all the relevant problem of feature selection is the identification of all strongly and weakly relevant attributes, especially for the time series classification and regression problems in the field of industrial application. The LBT forecasting is just the typical regression problem. Each label or regression target objectively has complex information association, which makes it more difficult to solve the problem of feature selection. To limit the number of irrelevant features, the tsfresh deploys the fresh algorithm, and the fresh stands for FeatuRe Extraction based on Scalable Hypothesis tests [29]. According to the importance of features to classification or regression problems, it filters available features in the early stage of machine learning process and controls the expected percentage of selected unrelated features. On a smaller scale, the filtering process is further divided into three stages: feature extraction, feature significance testing, and multiple test procedure. After this process, only a small part of the features that are relevant enough are retained; most uncorrelated or weakly correlated features can be removed, including the column of NaN (not a number) value. This module will remove all NaN values and select only the relevant features next. Finally, the filtered feature data file is exported to provide more feature support for the DNN computing architecture.

### 4.4. Deep Learning for Liner Berthing Time Prediction with Partial Feature Extraction

After critical feature extraction of the liner berthing and handling operational log, the definition of DNN computing architecture is made explicitly to drive the configuration, deployment, and execution of CTO-LGC. The QRM-LBT including the operational records of five years are marked as four data sets. Above all, the running log of YA and YB is defined as the quayside running mirror for liner berthing time with the log of two years (QRM-LBT-LTW), which includes 2686 records. In the second place, the running log of YA, YB, and YC is called after the quayside running mirror for liner berthing time with the log of three years (QRM-LBT-LTH) that covers 4036 items. Thirdly, the running log of YA, YB, YC, and YD is denominated the quayside running mirror for liner berthing time with the log of four years (QRM-LBT-LFO) that includes 6597 records. Lastly, the running log of YA, YB, YC, YD, and YE is regarded as the quayside running mirror for liner berthing time with the log of five years (QRM-LBT-LFI) that contain 9433 items.

For any of the above data sets, the whole is divided into three parts that are train subset, validation one, and test one, and the proportions of the three all are 80%, 16.82%, and 3.18%, respectively. Consequently, the LBT of 86, 129, 210, and 300 liners are going to be predicted that are about one week, two weeks, three weeks, and a month's worth of calling container ships for domestic trade routes. That is to say, the CTO-NPFC can make an intelligent decision support for the logistics service program of one week, two weeks, three weeks, and a month in this case, especially for berth allocation plan. The weekly and monthly berth allocation plan is a very important foundation, basis, and reference for PDP-SAD at container terminals. Meanwhile, by utilizing the tsfresh, we focus on the indicator of the actual operation time of ship (AOTS), which is directly related to LBT, to extract two types of critical features. One is the absolute energy of the time series that only has one secondary indicator, and the other is Mexican hat wavelet that covers four secondary indicators. The combination of the critical features and QRM-LBT is imported into the DLM-CCA to execute LBT forecasting.

The DLM-CCA adopts the sequential model that is a linear stack of multiple network layers. To make the CTO-NPFC more concrete, the DNN computing architecture is constructed by a four-layer network including LSTM, Gaussian Noise, bidirectional GRU, and dense network layer. Moreover, the number of artificial neural cells in each layer is distinctly different from each other. Meanwhile, the step size of the supervisory sequence is two, the training epoch of model is 60, and the batch size is 32. The loss function of mean absolute error (MAE), which is commonly used for regression problems, is applied to train and guide the generation of the DNN model. The loss function of MAE, root mean squared error (RMSE), and *R*-square is used for DNN model evaluation together, and the *R* is the coefficient of determination of DNN model. Theoretically speaking, the MAE and RMSE are expected to be close to zero, and the *R*-square is supposed to be approaching one.

**Figure 4:**
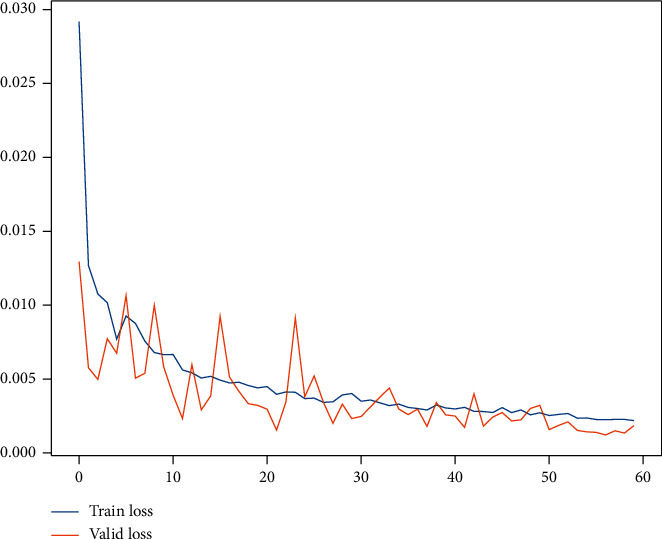
Typical DNN model training loss curves for QRM-LBT-LFI by partial feature extraction.

We set the different random number seeds for DNN architecture and execute 100 times for the data set of QRM-LBT-LTW, QRM-LBT-LTH, QRM-LBT-LFO, and QRM-LBT-LFI, respectively. The consuming time of each experiment is all between 35 seconds and 85 ones or so, and the time taken varies depending on the size of the data set. For the four data sets, the MAE of the train set is between 0.0022 and 0.0056, and the MAE of the validation set is between 0.0009 and 0.0058, which both testify to the generation and training of DNN model that have good performance because the two are pretty close to zero. The typical DNN model training loss curves can be illustrated by Figure 4 with partial feature extraction, which only takes the data set of QRM-LBT-LFI as an example due to limited length.

**Figure 5:**
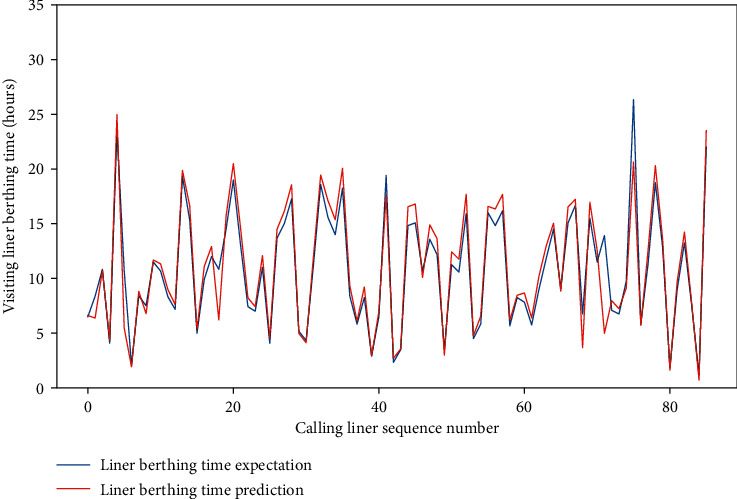
A comparison of LBT predictors with real values for QRM-LBT-LTW by partial features.

**Figure 6:**
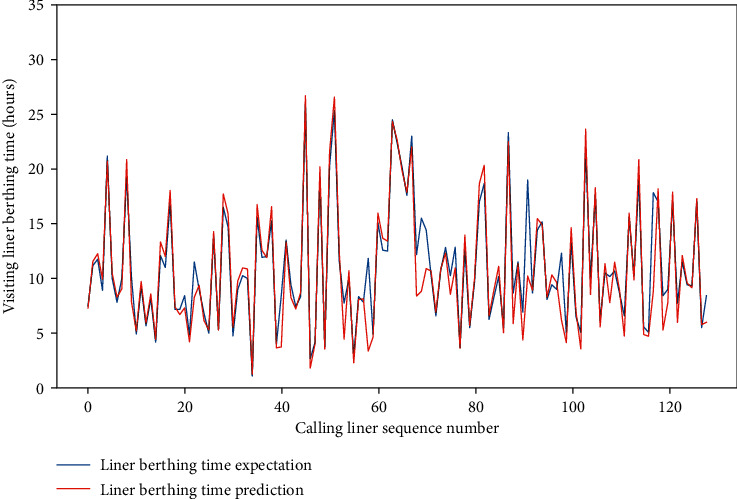
A comparison of LBT predictors with real values for QRM-LBT-LTH by partial features.

**Figure 7:**
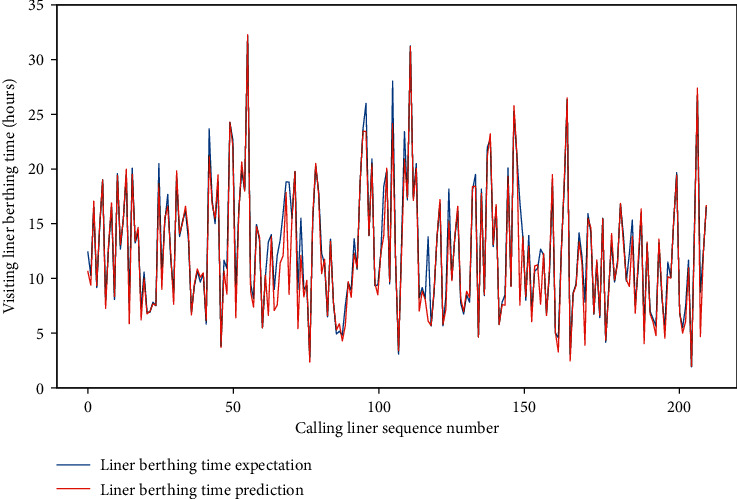
A comparison of LBT predictors with real values for QRM-LBT-LFO by partial features.

**Figure 8:**
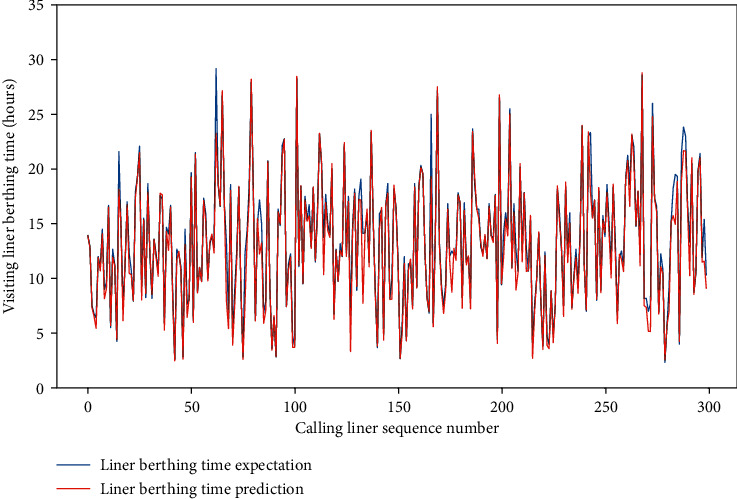
A comparison of LBT predictors with real values for QRM-LBT-LFI by partial features.

Meanwhile, for the four data sets, the MAE of the test set is approximately between 0.659 and 1.281, and the RMSE of the test set is between 1.206 and 2.001, and the *R*-square of the test set is approximately between 0.857 and 0.954. The comparison of typical LBT prediction results with real values for the four data sets can be showed in Figures 5–8, respectively. In total, the DNN model with partial feature extraction has given us some impressive performance improvements compared with the DNN model without feature extraction, which has been discussed in our previous studies [35].

### 4.5. Deep Learning for Liner Berthing Time Prediction with Complete Feature Extraction

**Figure 9:**
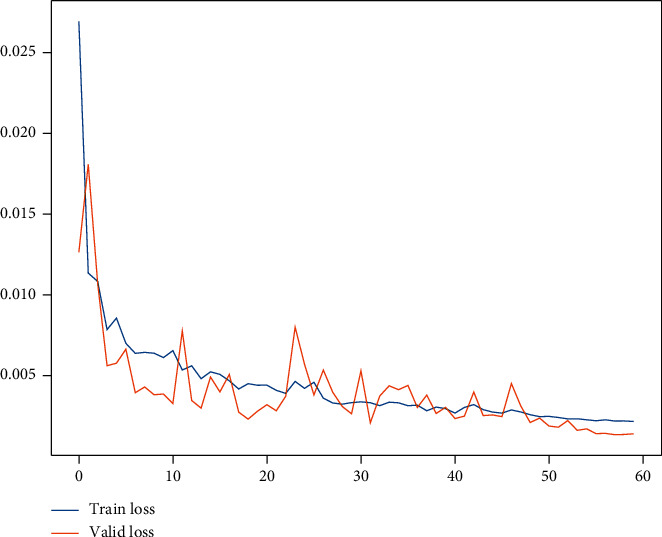
Typical DNN model training loss curves for QRM-LBT-LFI by complete feature extraction.

In Section 4.4, the feature extraction plays an important role in the prediction of LBT. Now, we further increase the depth and breadth of feature extraction. By the tsfresh, we focus on the indicator of AOTS and LBT simultaneously to extract the critical features of the absolute energy of the time series and the Mexican hat wavelet, which add the total of 10 secondary indicators. Through the same DNN model, we also execute 100 times for the data set of QRM-LBT-LTW, QRM-LBT-LTH, QRM-LBT-LFO, and QRM-LBT-LFI apart. The consuming time of each experiment is all about between 40 seconds and 93 ones, and the time taken varies depending on the size of the data set as well. For the four data sets, the MAE of the train set is between 0.0021 and 0.0041, and the MAE of the validation set is about between 0.0010 and 0.0047. Relative to the partial features' extraction, the progress of two is not particularly obvious. The representative DNN model training loss curves may be demonstrated by Figure 9 with complete feature extraction, which only takes the data set of QRM-LBT-LFI for example due to limited length too.

**Figure 10:**
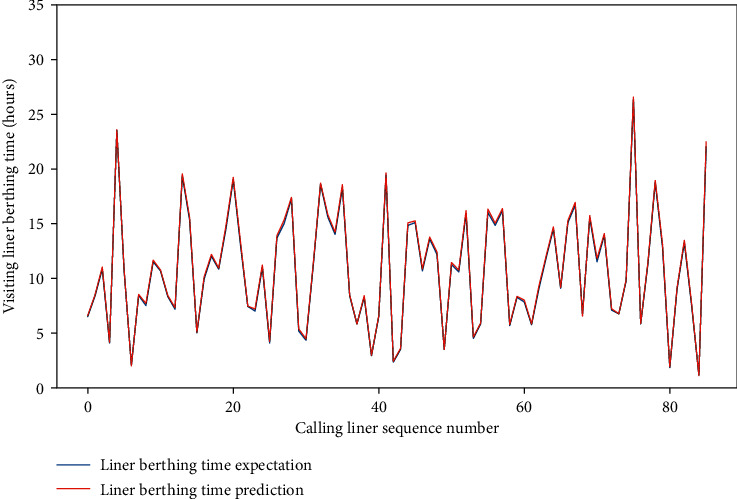
A comparison of LBT predictors with real values for QRM-LBT-LTW by complete features.

**Figure 11:**
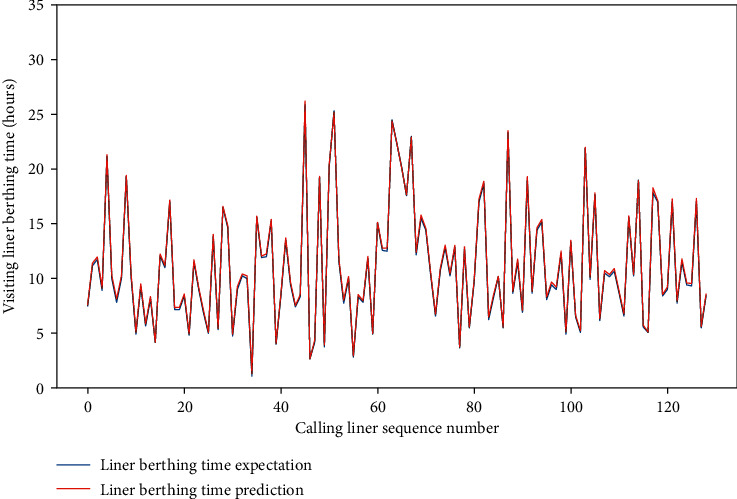
A comparison of LBT predictors with real values for QRM-LBT-LTH by complete features.

**Figure 12:**
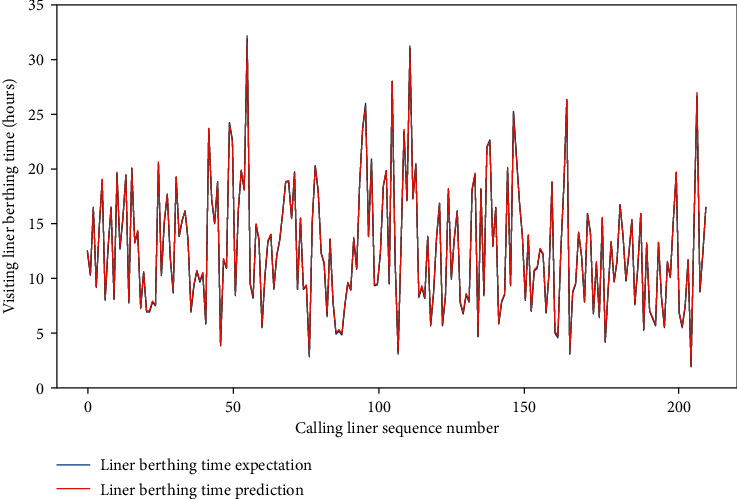
A comparison of LBT predictors with real values for QRM-LBT-LFO by complete features.

**Figure 13:**
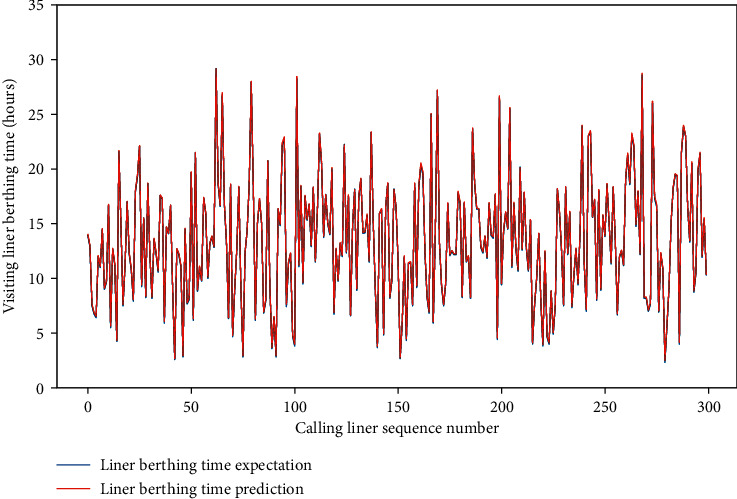
A comparison of LBT predictors with real values for the QRM-LBT-LFI by complete features.

At the same time, the MAE of the test set is between 0.029 and 0.331, and the RMSE of the test set is between 0.038 and 0.343 or so, and the *R*-square of the test set is approximately 0.999. All three improve significantly compared to the situations in partial feature extraction. The comparison of typical LBT prediction results with real values for the four data sets can be demonstrated by Figures 10–13 apart.

By contrast, there has been further performance improvement on the DLM-CCA with complete feature extraction compared with the one with partial feature extraction. Through the above experimental results, it is concluded that the DLM-CCA with the complete features can predict the LBT excellently. It means that the LBT can be basically confirmed once the berthing order is determined roughly. This is very beneficial to the task scheduling and resource allocation of the CTHS.

### 4.6. Forecasting Performance Evaluation of Liner Berthing Time

From a qualitative point of view, the DLM-CCA shows the prominent predictive function, and we will make further quantitative analysis on its forecasting performance now. Clearly, the prediction error of LBT is what we are most interested in. For two different treatments of partial feature extraction and complete feature extraction, we will discuss them each in turn and then compare and analyze the differences between the two. Now, we present comprehensive performance profiles of LBT prediction deviation, which is showed in the eight statistical tables listed in Tables 2–9. The former four tables are aiming at the partial feature extraction from the data set of QRM-LBT-LTW, QRM-LBT-LTH, QRM-LBT-LFO, and QRM-LBT-LFI apart, and the latter four ones are specific to the complete feature extraction from the same four data sets. It provides a critical insight into the deep learning forecasting performance of the DLM-CCA.

In order to facilitate the statistical analysis of the specific prediction deviation of LBT, we process and calculate the absolute value of the differences between the predicted value and the real value for the indicator of LBT. In view of the different distribution range of LBT prediction deviation using partial feature extraction and complete feature extraction, we set the LBT deviation levels on the basis of keeping corresponding relations for comparison. For the experimental results obtained by partial features, we first define [0, 0.5] and [0.5, 1] as the first and second levels of scale contrast and then divide the LBT prediction deviation into another five levels according to the time interval of one hour. For the results obtained by complete extraction, we divide the prediction deviation of LBT into six levels with an interval of 0.1 hours by taking the deviation range [0, 0.1] as the first level.

For one thing, the quantity of LBT forecasting error between 0 and 1 accounts for the largest proportion with partial feature extraction, which is more than 50% for all the data sets and increases as the time span grows to 82.073%. Meanwhile, the proportion of LBT prediction deviation in [0, 5] is more than 96% for any data set. The prediction accuracy with partial features is much better than the pure DNN model whose prediction deviation in [0, 5] is only approximately 73% [35].

Furthermore, the quantity of LBT forecasting deviation between 0 and 0.1 accounts for the largest proportion with complete feature extraction, and it reaches up to 47.151%, 51.574%, 62.276%, and 71.650% for the four data sets. Moreover, the proportion of LBT prediction deviation less than or equal to 0.5 hours is more than 99.8% for any one data set. The prediction accuracy with complete features is improved tenfold versus the one by partial features, which indicates the DLM-CCA possesses the outstanding predictive performance. However, it is worth mentioning that the SD value increases visibly with the rise of prediction quantity at the same prediction deviation level of [0, 0.1] and (0.1, 0.2] both.

In addition, we set three indexes including MAE, RMSE, and *R*-square to evaluate the performance of the DNN model. Tables 10–12 are the evaluating indicator results by partial features extraction, and Tables 13–15 are the ones with complete features extraction. By comparing two sets of data table collections, it is concluded that the latter is also superior to the former dramatically whether for MAE or for RMSE. Especially for the index of *R*-square, it is very close to the theoretical optimal value of 1 while adopting complete features extraction.

A final note about the DLM-CCA is that its computational cost and consume time are easy to accept. The experimental platform is very common as described in Section 4.1. Both of Tables 16 and 17 show the total running time (TRT) analysis of the DNN model based on partial features and complete features, respectively. In either case, we can get the LBT forecasting weekly or monthly within 95 seconds, and there are little differences in LBT forecasting performance. It is a very prominent performance both in theory and in practice.

Through the above experiments and evaluations, it is concluded that the CTO-NPFC provides a rational, agile, flexible, efficient, and robust intelligent decision support solutions to the PDP-SAD of CTHS. The CTO-NPFC trilogy is mainly the container terminal physical logistics service-oriented abstraction and automation that is just CTO-LGC, critical feature extraction of CTO-LGC running mirror, and pattern recognition and regression analysis with deep learning for the combination of filtering critical features and CTO-LGC running mirror. It is supposed to propose a referenced theoretical framework and practical solution for the exploration and exploitation of RGT-UIF-PoC in the given field of complex logistics hubs.

## 5. Conclusions

This paper focuses on the automation and intelligence of CTO-LGC by the combination, integration, and fusion of computational logistics and deep learning and proposes the conceptual framework of CTO-NPFC to solve the PDP-SAD at container terminals. The thinking of CTO-NPFC seems to be similar to digital twin and cyber-physical system, but there are differences substantially among the three. In the CTO-NPFC, the CTO-LGC in the physical world is combined with DNN computation in the cyberspace, and the neural computation drives physical computation and the computation under different physical sizes are combined to complement each other, which conceives, designs, implements, deploys, drives, evaluates, and optimizes the operation of CTHS, especially in the context of low carbon emissions and sustainable development. The CTO-NPFC is supposed to achieve and fulfill the mechanization, automation, and intelligence of CTO-LGC. Moreover, the synergy between computational logistics and deep learning provides an insight, perspective, paradigm, and practice to explore and exploit the nature, intension, extension, and application of computation in the domain of CLS and complex operating systems.

## Figures and Tables

**Table 1:** Distribution characteristic value of LBT for domestic trade routes in China (unit: hours).

Year	Quantity of liners	Minimum of LBT	Maximum of LBT	Mean of LBT	Median of LBT	Mode of LBT	SD of LBT	Variance of LBT
YA	1422	0.667	34.750	10.196	9.500	8.500	4.818	23.217
YB	1264	0.750	34.833	9.617	9.083	7.000	4.744	22.506
YC	1350	0.750	32.750	10.221	9.417	8.667	4.950	24.503
YD	2561	1.000	34.000	12.137	11.667	7.833	5.560	30.909
YE	2836	0.583	40.833	12.248	11.750	10.167	5.624	31.629

**Table 2:** Prediction deviation profile of liner berthing time for QRM-LBT-LTW by partial features.

Prediction deviation (hours)	Minimum of liners	Maximum of liners	Mean of liners	Median of liners	Mode of liners	SD of liners	Variance of liners	Quantitative proportion of liners (%)
[0, 0.5]	17.000	34.000	25.440	25.000	26.000	3.864	14.926	29.582
(0.5, 1]	19.000	28.000	23.590	24.000	23.000	1.795	3.222	27.430
(1, 2]	20.000	43.000	31.390	31.000	31.000	4.361	19.018	36.500
(2, 3]	0.000	3.000	0.890	1.000	1.000	0.527	0.278	1.035
(3, 4]	0.000	1.000	0.690	1.000	1.000	0.463	0.214	0.802
(4, 5]	1.000	2.000	1.070	1.000	1.000	0.255	0.065	1.244
(5, +∞]	2.000	3.000	2.930	3.000	3.000	0.255	0.065	3.407

**Table 3:** Prediction deviation profile of liner berthing time for QRM-LBT-LTH by partial features.

Prediction deviation (hours)	Minimum of liners	Maximum of liners	Mean of liners	Median of liners	Mode of liners	SD of liners	Variance of liners	Quantitative proportion of liners (%)
[0, 0.5]	34.000	57.000	49.620	50.000	52.000	3.638	13.236	38.465
(0.5, 1]	29.000	43.000	33.930	34.000	33.000	2.471	6.105	26.302
(1, 2]	24.000	37.000	29.430	29.000	30.000	2.543	6.465	22.814
(2, 3]	5.000	8.000	5.560	5.000	5.000	0.668	0.446	4.310
(3, 4]	3.000	5.000	4.460	5.000	5.000	0.607	0.368	3.458
(4, 5]	1.000	1.000	1.000	1.000	1.000	0.000	0.000	0.775
(5, +∞]	5.000	5.000	5.000	5.000	5.000	0.000	0.000	3.876

**Table 4:** Prediction deviation profile of liner berthing time for QRM-LBT-LFO by partial features.

Prediction deviation (hours)	Minimum of liners	Maximum of liners	Mean of liners	Median of liners	Mode of liners	SD of liners	Variance of liners	Quantitative proportion of liners (%)
[0, 0.5]	108.000	134.000	126.920	128.000	129.000	4.724	22.314	60.438
(0.5, 1]	33.000	68.000	44.110	43.000	43.000	6.107	37.298	21.005
(1, 2]	15.000	25.000	18.540	18.000	18.000	1.717	2.948	8.829
(2, 3]	6.000	8.000	7.310	7.000	7.000	0.674	0.454	3.481
(3, 4]	4.000	7.000	5.660	6.000	6.000	0.533	0.284	2.695
(4, 5]	2.000	4.000	3.010	3.000	3.000	0.500	0.250	1.433
(5, +∞]	4.000	5.000	4.450	4.000	4.000	0.498	0.248	2.119

**Table 5:** Prediction deviation profile of liner berthing time for QRM-LBT-LFI by partial features.

Prediction deviation (hours)	Minimum of liners	Maximum of liners	Mean of liners	Median of liners	Mode of liners	SD of liners	Variance of liners	Quantitative proportion of liners (%)
[0, 0.5]	160.000	197.000	179.190	178.000	173.000	8.659	74.974	59.730
(0.5, 1]	58.000	76.000	67.030	68.000	68.000	4.385	19.229	22.343
(1, 2]	22.000	42.000	30.150	30.000	28.000	4.410	19.448	10.050
(2, 3]	7.000	10.000	8.630	9.000	9.000	0.702	0.493	2.877
(3, 4]	6.000	7.000	6.960	7.000	7.000	0.196	0.038	2.320
(4, 5]	2.000	3.000	2.520	3.000	3.000	0.500	0.250	0.840
(5, +∞]	5.000	6.000	5.520	6.000	6.000	0.500	0.250	1.840

**Table 6:** Prediction deviation profile of liner berthing time for QRM-LBT-LTW by complete features.

Prediction deviation (hours)	Minimum of liners	Maximum of liners	Mean of liners	Median of liners	Mode of liners	SD of liners	Variance of liners	Quantitative proportion of liners (%)
[0, 0.1]	0.000	82.000	40.550	41.500	16.000	24.026	577.248	47.151
(0.1, 0.2]	4.000	54.000	28.740	28.000	19.000	13.197	174.172	33.419
(0.2, 0.3]	0.000	55.000	11.970	6.000	2.000	12.467	155.429	13.919
(0.3, 0.4]	0.000	45.000	4.060	1.000	0.000	8.335	69.476	4.721
(0.4, 0.5]	0.000	17.000	0.580	0.000	0.000	2.426	5.884	0.674
(0.5, +∞]	0.000	3.000	0.100	0.000	0.000	0.436	0.190	0.116

**Table 7:** Prediction deviation profile of liner berthing time for QRM-LBT-LTH by complete features.

Prediction deviation (hours)	Minimum of liners	Maximum of liners	Mean of liners	Median of liners	Mode of liners	SD of liners	Variance of liners	Quantitative proportion of liners (%)
[0, 0.1]	8.000	116.000	66.530	75.500	38.000	34.747	1207.329	51.574
(0.1, 0.2]	8.000	85.000	43.830	43.000	73.000	23.163	536.541	33.977
(0.2, 0.3]	0.000	64.000	14.590	7.000	3.000	15.359	235.902	11.310
(0.3, 0.4]	0.000	16.000	3.210	2.000	2.000	3.080	9.486	2.488
(0.4, 0.5]	0.000	5.000	0.640	0.000	0.000	0.855	0.730	0.496
(0.5, +∞]	0.000	3.000	0.200	0.000	0.000	0.490	0.240	0.155

**Table 8:** Prediction deviation profile of liner berthing time for QRM-LBT-LFO by complete features.

Prediction deviation (hours)	Minimum of liners	Maximum of liners	Mean of liners	Median of liners	Mode of liners	SD of liners	Variance of liners	Quantitative proportion of liners (%)
[0, 0.1]	12.000	196.000	130.780	143.000	191.000	55.869	3121.332	62.276
(0.1, 0.2]	7.000	161.000	67.960	58.000	29.000	49.525	2452.738	32.362
(0.2, 0.3]	1.000	46.000	8.8400	5.000	4.000	9.047	81.854	4.210
(0.3, 0.4]	0.000	6.000	1.720	1.000	1.000	1.415	2.002	0.819
(0.4, 0.5]	0.000	7.000	0.490	0.000	0.000	0.933	0.870	0.233
(0.5, +∞]	0.000	3.000	0.210	0.000	0.000	0.668	0.446	0.100

**Table 9:** Prediction deviation profile of liner berthing time for QRM-LBT-LFI by complete features.

Prediction deviation (hours)	Minimum of liners	Maximum of liners	Mean of liners	Median of liners	Mode of liners	SD of liners	Variance of liners	Quantitative proportion of liners (%)
[0, 0.1]	6.000	294.000	214.950	237.000	288.000	80.353	6456.548	71.650
(0.1, 0.2]	6.000	236.000	77.4500	60.000	12.000	68.301	4664.968	25.817
(0.2, 0.3]	0.000	100.000	7.030	1.000	0.000	16.150	260.809	2.343
(0.3, 0.4]	0.000	14.000	0.500	0.000	0.000	2.062	4.250	0.167
(0.4, 0.5]	0.000	6.000	0.060	0.000	0.000	0.597	0.356	0.020
(0.5, +∞]	0.000	1.000	0.010	0.000	0.000	0.100	0.010	0.003

**Table 10:** MAE evaluation index profiles of liner berthing time prediction experiment by partial features.

QRM-LBT	Minimum of MAE	Maximum of MAE	Mean of MAE	Median of MAE	SD of MAE	Variance of MAE
QRM-LBT-LTW	0.998	1.281	1.124	1.128	0.060	0.004
QRM-LBT-LTH	1.126	1.213	1.167	1.168	0.017	0.000
QRM-LBT-LFO	0.826	0.856	0.840	0.839	0.006	0.000
QRM-LBT-LFI	0.659	0.797	0.720	0.719	0.032	0.001

**Table 11:** RMSE evaluation index profiles of liner berthing time prediction experiment by partial features.

QRM-LBT	Minimum of RMSE	Maximum of RMSE	Mean of RMSE	Median of RMSE	SD of RMSE	Variance of RMSE
QRM-LBT-LTW	1.672	1.773	1.716	1.713	0.023	0.001
QRM-LBT-LTH	1.905	2.001	1.952	1.953	0.016	0.000
QRM-LBT-LFO	1.593	1.684	1.640	1.640	0.015	0.000
QRM-LBT-LFI	1.206	1.333	1.268	1.267	0.029	0.001

**Table 12:** *R*-square evaluation index profiles of liner berthing time prediction experiment by partial features.

QRM-LBT	Minimum of *R*-square	Maximum of *R*-square	Mean of *R*-square	Median of *R*-square	SD of *R*-square	Variance of *R*-square
QRM-LBT-LTW	0.886	0.899	0.893	0.893	0.003	0.000
QRM-LBT-LTH	0.857	0.870	0.864	0.864	0.002	0.000
QRM-LBT-LFO	0.912	0.921	0.917	0.916	0.002	0.000
QRM-LBT-LFI	0.943	0.954	0.949	0.949	0.002	0.000

**Table 13:** MAE evaluation index profiles of liner berthing time prediction experiment by complete features.

QRM-LBT	Minimum of MAE	Maximum of MAE	Mean of MAE	Median of MAE	SD of MAE	Variance of MAE
QRM-LBT-LTW	0.048	0.331	0.127	0.109	0.064	0.004
QRM-LBT-LTH	0.054	0.216	0.116	0.103	0.044	0.002
QRM-LBT-LFO	0.039	0.179	0.093	0.090	0.034	0.001
QRM-LBT-LFI	0.029	0.187	0.076	0.070	0.038	0.001

**Table 14:** RMSE evaluation index profiles of liner berthing time prediction experiment by complete features.

QRM-LBT	Minimum of RMSE	Maximum of RMSE	Mean of RMSE	Median of RMSE	SD of RMSE	Variance of RMSE
QRM-LBT-LTW	0.063	0.343	0.144	0.132	0.063	0.004
QRM-LBT-LTH	0.069	0.231	0.138	0.127	0.040	0.002
QRM-LBT-LFO	0.060	0.188	0.110	0.105	0.030	0.001
QRM-LBT-LFI	0.038	0.195	0.086	0.080	0.037	0.001

**Table 15:** *R*-square evaluation index profiles of liner berthing time prediction experiment by complete features.

QRM-LBT	Minimum of *R*-square	Maximum of *R*-square	Mean of *R*-square	Median of *R*-square	SD of *R*-square	Variance of *R*-square
QRM-LBT-LTW	0.996	1.000	0.999	0.999	0.001	0.000
QRM-LBT-LTH	0.998	1.000	0.999	0.999	0.000	0.000
QRM-LBT-LFO	0.999	1.000	1.000	1.000	0.000	0.000
QRM-LBT-LFI	0.999	1.000	1.000	1.000	0.000	0.000

**Table 16:** Total running time profiles of liner berthing time prediction by partial features (unit: seconds).

QRM-LBT	Minimum of TRT	Maximum of TRT	Mean of TRT	Median of TRT	Mode of TRT	SD of TRT	Variance of TRT
QRM-LBT-LTW	35.105	42.439	36.218	35.800	35.623	1.281	1.641
QRM-LBT-LTH	43.787	47.906	44.795	44.640	43.929	0.711	0.506
QRM-LBT-LFO	58.764	66.331	59.516	59.333	59.045	0.855	0.732
QRM-LBT-LFI	76.880	83.270	78.737	78.491	78.436	1.100	1.210

**Table 17:** Total running time profiles of liner berthing time prediction by complete features (unit: seconds).

QRM-LBT	Minimum of TRT	Maximum of TRT	Mean of TRT	Median of TRT	Mode of TRT	SD of TRT	Variance of TRT
QRM-LBT-LTW	40.972	47.047	43.288	42.956	43.882	1.374	1.887
QRM-LBT-LTH	45.972	62.255	53.382	53.805	52.710	2.907	8.451
QRM-LBT-LFO	61.907	81.319	64.215	63.293	62.939	3.063	9.384
QRM-LBT-LFI	80.034	92.435	83.489	82.790	81.739	2.407	5.795

## Data Availability

The data used to support the findings of this study are included within the article.
